# Circadian Modulation of Gene Expression, but not Glutamate Uptake, in Mouse and Rat Cortical Astrocytes

**DOI:** 10.1371/journal.pone.0007476

**Published:** 2009-10-15

**Authors:** Christian Beaulé, Adrienne Swanstrom, María Juliana Leone, Erik D. Herzog

**Affiliations:** Department of Biology, Washington University, Saint Louis, Missouri, United States of America; Yale School of Medicine, United States of America

## Abstract

**Background:**

Circadian clocks control daily rhythms including sleep-wake, hormone secretion, and metabolism. These clocks are based on intracellular transcription-translation feedback loops that sustain daily oscillations of gene expression in many cell types. Mammalian astrocytes display circadian rhythms in the expression of the clock genes *Period1* (*Per1*) and *Period2* (*Per2*). However, a functional role for circadian oscillations in astrocytes is unknown. Because uptake of extrasynaptic glutamate depends on the presence of *Per2* in astrocytes, we asked whether glutamate uptake by glia is circadian.

**Methodology/Principal Findings:**

We measured glutamate uptake, transcript and protein levels of the astrocyte-specific glutamate transporter, *Glast*, and the expression of *Per1* and *Per2* from cultured cortical astrocytes and from explants of somatosensory cortex. We found that glutamate uptake and *Glast* mRNA and protein expression were significantly reduced in *Clock/Clock, Per2-* or *NPAS2*-deficient glia. Uptake was augmented when the medium was supplemented with dibutyryl-cAMP or B27. Critically, glutamate uptake was not circadian in cortical astrocytes cultured from rats or mice or in cortical slices from mice.

**Conclusion/Significance:**

We conclude that glutamate uptake levels are modulated by CLOCK, PER2, NPAS2, and the composition of the culture medium, and that uptake does not show circadian variations.

## Introduction

Circadian rhythmicity is observed at all levels of physiology and behavior. In mammals, the molecular mechanism responsible for the generation of circadian oscillations arises from interacting transcription-translation feedback loops involving a set of key clock genes [1], [2]. The transcription factors *Clock* and *Bmal1* induce the expression of the *Period* and *Cryptochrome* genes. *Period* and *Cryptochrome* gene products then feed back to suppress CLOCK/BMAL1 activity, thus inhibiting their own transcription. These loops are expressed in many cell types where they regulate rhythmicity in most aspects of physiology [3]. For example, cortical astrocytes in culture display circadian rhythms in the expression of the clock genes *Per1* and *Per2* [4], [5]and in extracellular ATP content [6]. The role of these molecular rhythms in glial function is unknown.

Mutations in key clock genes disrupt overt circadian rhythms and have been associated with phenotypes such as obesity, decreased longevity, increased sensitivity to substances of abuse, depression and other cognitive impairments [1], [3], [7]. For example, the dominant-negative, antimorphic mutation in the *Clock* gene is responsible for the gradual loss of locomotor rhythms of *Clock/Clock* mice housed in constant darkness [8] and mania-like behaviors [9]. Deletion of the basic helix-loop-helix PAS motif in the neuronal PAS domain protein 2 (*NPAS2*) gene (an analog of *Clock)* leads to subtle circadian locomotor deficits and disrupts learning and memory and sleep architecture [10], [11]. Loss of both NPAS2 and CLOCK abolishes circadian rhythms in locomotion [12] and in peripheral organs [13]. Null mutations of both the *Per1* and *Per2* genes (*Per1^ldc^* and *Per2^ldc^*) abolish circadian locomotor rhythms [14].

The best evidence that glia participate in circadian behaviors comes from *Drosophila* where activity of the glia-specific gene, *ebony*, is required for normal circadian rhythms in locomotion [15]. In mammals, one study has examined the role of circadian clock genes in astrocyte physiology. *Per2^Brdm1^* mutant mice exhibit a phenotype similar to human alcoholism with elevated extracellular levels of glutamate in the brain, decreased glutamate uptake by cortical astrocytes and reduced expression of the high-affinity glutamate-aspartate transporter GLAST (EAAT1) [16]. Based on these results, we hypothesized that GLAST-dependent glutamate uptake by astrocytes is regulated by components of the molecular circadian clock. To test this hypothesis, we measured glutamate uptake in cultured astrocytes of different genotypes, in cortical slices and across time. Our results show that glutamate uptake by glia is regulated by the *Per2*, *Clock* and *NPAS2* genes, but that it is not circadian.

## Results

### The Clock and Per2 genes regulate glutamate uptake and GLAST levels in glia

**Figure 1:**
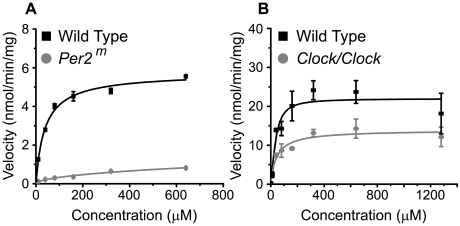
Glutamate uptake depends on the *Clock* and *Per2* genes. Dose–response curves for glutamate uptake were generated comparing wild-type astrocyte cultures and either *Clock/Clock* or *Per2^m^* mutant astrocytes. A, Glutamate uptake was significantly reduced in astrocytes derived from *Clock/Clock* mutants compared to wild-type (+/+) glia (n = 3 cultures per concentration, mean±SEM). B, Glutamate uptake was significantly reduced in astrocytes derived from *Per2^m^* mutants compared to wild-type (+/+) glia (n = 3 per concentration, mean±SEM).

To confirm and expand the previous observation that the Per2 gene modulates glutamate uptake in glia [16], we measured glutamate uptake in astrocytes cultured from mice of different circadian genotypes. In agreement with [16], we found that the lack of the *Per2* gene (*Per2^m^*) significantly reduced glutamate uptake (Fig. 1A). Glutamate uptake in *Per2^m^* glia was significantly lower than wild type at all concentrations tested (F_(1,28)_ = 209.2, p<0.0001). We also found that the homozygous *Clock* mutation diminished astroglial glutamate uptake (Fig. 1B). Maximal uptake velocity, Vmax, was significantly reduced in *Clock/Clock* compared to homozygous PER2::LUC (+/+) astrocytes (13.0±1.1 *vs.* 21.4±2.3 nmol/min/mg respectively, p<0.01). Affinity of the transporter, Km, was not affected. Lower uptake by *Clock/Clock* astrocytes replicated in four independent experiments. These results indicate that glutamate uptake depends on PER2 and CLOCK expression.

**Figure 2:**
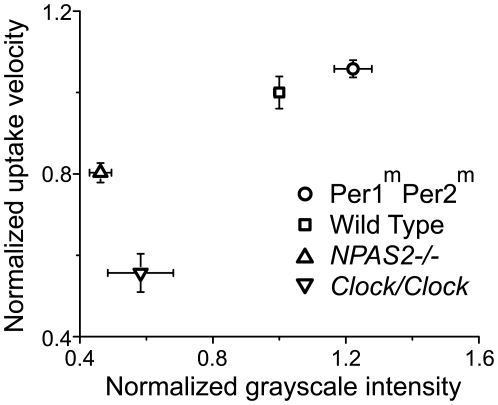
Higher glutamate uptake is associated with higher GLAST immunofluorescence. Scatter plot shows the relationship between glutamate uptake and grayscale intensity of GLAST immunofluorescence. Data normalized to wild type levels (wild type: n = 7; *NPAS2−/−*: n = 6; *Per1^m^Per2^m^*: n = 4; *Clock/Clock*: n = 2, mean±SEM).

We next tested whether the reduced glutamate uptake in *Clock/Clock* astrocytes correlates with a reduction in the levels of the glutamate transporter, *Glast*. We found using qRT-PCR that the *Clock/Clock* mutation reduced *Glast* mRNA levels by 2.5-fold (61% decrease, p<0.001, n = 2 independent experiments performed in triplicate). The *Clock/Clock* mutation also reduced GLAST protein immunofluorescence by approximately 70% (p<0.0001, n = 2 independent experiments performed in triplicate). In addition, *NPAS2*-deficient, but not *Per1^m^Per2^m^* mutant, astrocytes showed reduced GLAST immunofluorescence (50% decrease compared to wild-type, F_(2,4)_ = 44, p<0.01, n = 3 and 2 cultures, respectively). These results indicated clock gene regulation of GLAST expression which correlated with glutamate uptake (Fig. 2).

#### Glutamate uptake is not circadian in mouse and rat cortical astrocytes

**Figure 3:**
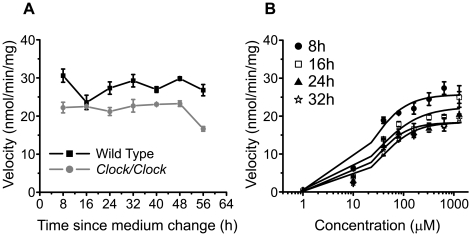
Glutamate uptake is not circadian in cultured astrocytes. A, Uptake was measured every 8 h after a full medium exchange in wild type (black line) or in *Clock/Clock* (gray line) mouse cortical glia (200 µM tritiated glutamate, n = 3 cultures per time point; mean±SEM). B, Glutamate uptake depended on glutamate concentration, but not circadian time, in rat astrocytes. Dose-response curves were generated every 8 h after a full medium exchange. Neither the maximal uptake velocity (Vmax) nor the concentration for half-maximal uptake (Km) varied with time of day (n = 3 cultures per time point at each glutamate concentration; mean±SEM).

We tested whether glutamate uptake is circadian in astrocytes cultured from wild-type and *Clock/Clock* mice and from rats. We found that uptake was significantly lower in *Clock/Clock* mutant glia compared to wild type (21.7±0.9 *vs.* 27.8±0.9 nmol/min/mg respectively, p<0.001), but that uptake did not vary over time (Fig. 3A). Similar results were found in rat and mouse astrocytes sampled every 4 h starting 12 h after medium change (data not shown). Next, we measured glutamate uptake in rat astrocytes as a function of time and of extracellular glutamate concentration to determine whether circadian modulation of glutamate uptake is dose-dependent. We found that Vmax was higher 8 h after a medium change, but no evidence for circadian modulation of maximal or half-maximal (Km) uptake (Fig. 3B).

#### Modulation of clock gene expression in glia

**Figure 4:**
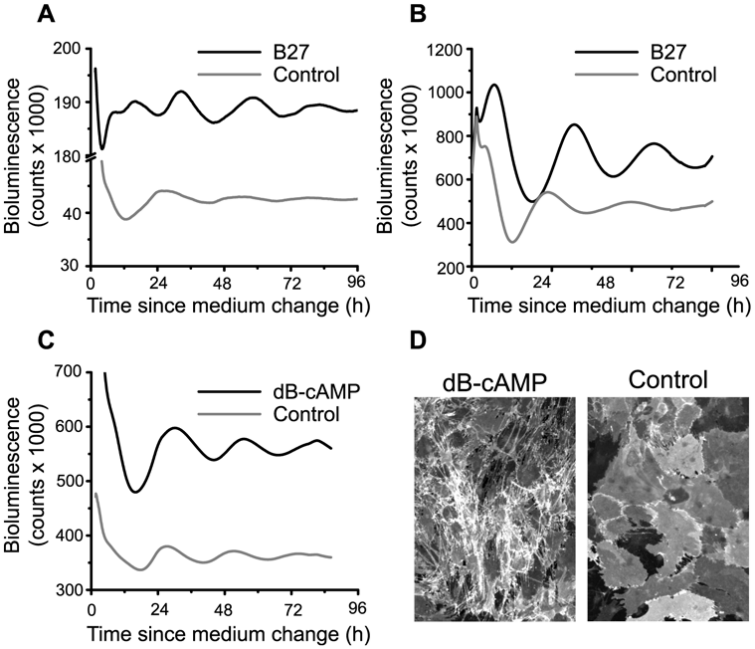
Circadian gene expression in astrocytes is modulated by culture conditions *in vitro*. A, Bioluminescence traces from rat *Per1::luc* cortical astrocytes. Supplementing the medium with B27 significantly increased *Per1*-driven bioluminescence levels and circadian rhythm amplitude (average of 2 cultures per traces). B, Bioluminescence traces from mouse PER2::LUC cortical glia showing higher bioluminescence levels and amplitude in the presence of B27 (average of 2 cultures per traces). C, Bioluminescence traces from rat cortical astrocytes showing that a 10-day treatment with dB-cAMP (250 µM) significantly increased *Per1*-driven bioluminescence levels and rhythm amplitude (average of 2 cultures per traces). D, Representative photomicrographs of GLAST immunofluorescence showing stellation of rat astrocytes induced by 250 µM dB-cAMP. Magnification = 10×.

We assayed whether circadian rhythms in astroglia were affected by the composition of the culture medium. We used two bioluminescent reporters of *Period* expression. Bioluminescence driven by *Per1::luc* reflects *Per1* promoter activity (transcription) whereas bioluminescence from PER2::LUC is a measure of PER2 protein levels (translation). We found that bioluminescence levels and rhythm amplitude for both *Per1::luc* (rat cortical glia) and PER2::LUC (mouse cortical glia) were increased in the presence of B27, a serum-free culture supplement used to promote cell survival [17] (Fig. 4A, 4B). Adding B27 (1X final concentration) to rat cortical astrocyte cultures increased bioluminescence levels more than four-fold (4.3±0.11, n = 4) and peak-to-trough levels two-fold (2.0±0.6, n = 4) after 24 h of recording (Fig. 4A). Adding the same concentration of B27 to mouse cortical astrocytes increased bioluminescence levels 1.5 fold (n = 2) and peak-to-trough levels more than two-fold (2.4 fold, n = 2, Fig. 4B). We hypothesized that factors in B27 might elevate clock gene expression in glia through cAMP, a strong regulator of circadian rhythmicity [18]. To test this hypothesis, we recorded glial bioluminescence rhythms for rat *Per1::luc* astrocytes in medium supplemented with 250 µM dibutyryl-cAMP (dB-cAMP) for 10 days prior to and during bioluminescence recording. We found that dB-cAMP increased *Per1::luc* bioluminescence levels 1.6-fold (n = 2) and peak-to-trough amplitude two-fold (n = 2) (Fig. 4C) and, consistent with previous reports [19], [20]induced stellation of glial processes (Fig. 4D). Thus, B27 or dB-cAMP can enhance *Period* gene expression and rhythm amplitude in cortical astrocytes.

#### Glutamate uptake depends on B27, and dB-cAMP, but not circadian time

**Figure 5:**
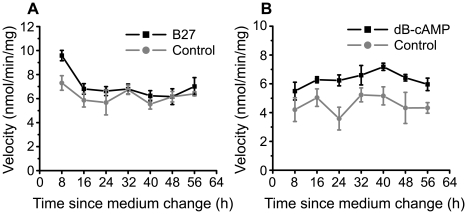
B27 or dB-cAMP as culture supplements do not induce a circadian rhythm in glutamate uptake. A, Uptake was measured in rat cortical astrocytes with (black line) or without B27 supplements (gray line) (500 µM tritiated glutamate, n = 3 cultures per time point; mean±SEM). B, A 10-day treatment of rat astrocytes with dB-cAMP (250 µM, black line) significantly increased overall glutamate uptake (500 µM tritiated glutamate), but did not induce circadian rhythmicity (n = 3 cultures per time point; mean±SEM).

Because we found that B27 and dB-cAMP increased circadian rhythmicity, we decided to test the hypothesis that B27 and dB-cAMP can induce circadian rhythms in glutamate uptake. We measured glutamate uptake in rat cortical astrocytes at 8 h intervals following a full medium change. Supplementing culture medium with B27 caused a significant 13% increase in glutamate uptake (p<0.05, Fig. 5A), but did not induce rhythmicity in uptake. Similarly, addition of 250 µM dB-cAMP increased glutamate import by an average of 39% (Fig. 5B, p<0.0001), but did not induce glutamate uptake rhythms. Finally, we performed qRT-PCR for the astrocyte-specific glutamate transporter, *Glast*. In two independent experiments, we failed to detect circadian variation in *Glast* mRNA in mouse cortical astrocytes cultured in B27-supplemented media (experiment 1, F_(6,8)_ = 1.59, p>0.05; experiment 2, F_(6,14)_ = 1.60, p>0.05).

#### Glutamate uptake is not circadian in cortical slices

**Figure 6:**
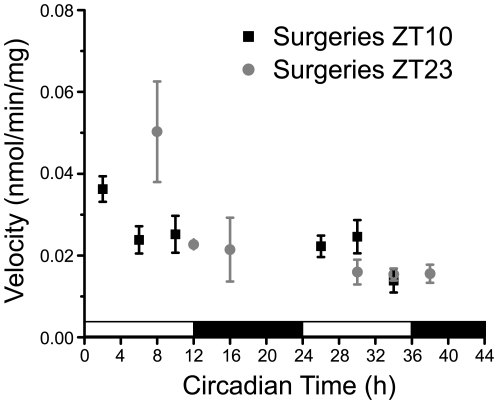
Glutamate uptake in slices of somatosensory cortex is not circadian. Explants were taken either 2 h before dusk (ZT 10, black squares) or 2 h before dawn (ZT 23, gray circles) and cultured for 36 h *in vitro* until 10-min uptake of 25 µM tritiated glutamate was measured. White and black bars on top of the X axis represent the projected subjective day and subjective night, respectively. (n = 3 slices per time point; mean±SEM).

**Figure 7:**
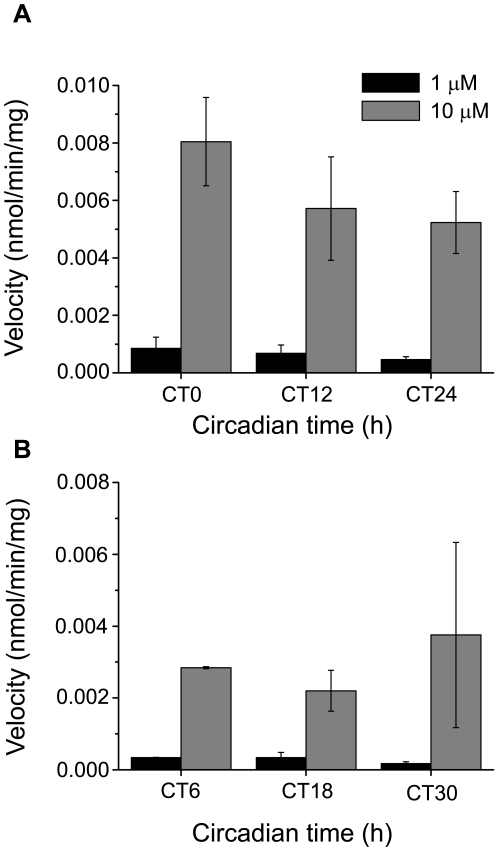
Glutamate uptake in mouse cortical slices is dose dependent, but not circadian. A–B, Two independent experiments showing that glutamate uptake is approximately 10 fold higher for a 10 µM glutamate concentration than for a 1 µM concentration. In addition, glutamate uptake did not show circadian variations at any concentration (mean±SEM). n = 3 for each time and concentration (A), n = 2 for each time and concentration (B).

To test whether the presence of neurons is required for the induction of rhythms in glutamate uptake, we measured uptake in mouse cortical slices over two consecutive days, starting 36 h after *in vitro* preparation. We measured at doses ranging from 1–25 µM glutamate based on the recent report that extracellular glutamate levels in the cortex fluctuate between 20–30 µM [21]. We found that uptake in cortical slices was augmented with higher glutamate loads, was higher on the first than the second day *in vitro* (F_(1,34)_ = 11.09, p<0.01) but not circadian at any dose or day *in vitro* (Fig. 6 and Fig. 7).

#### Specificity of the glutamate uptake assay

**Figure 8:**
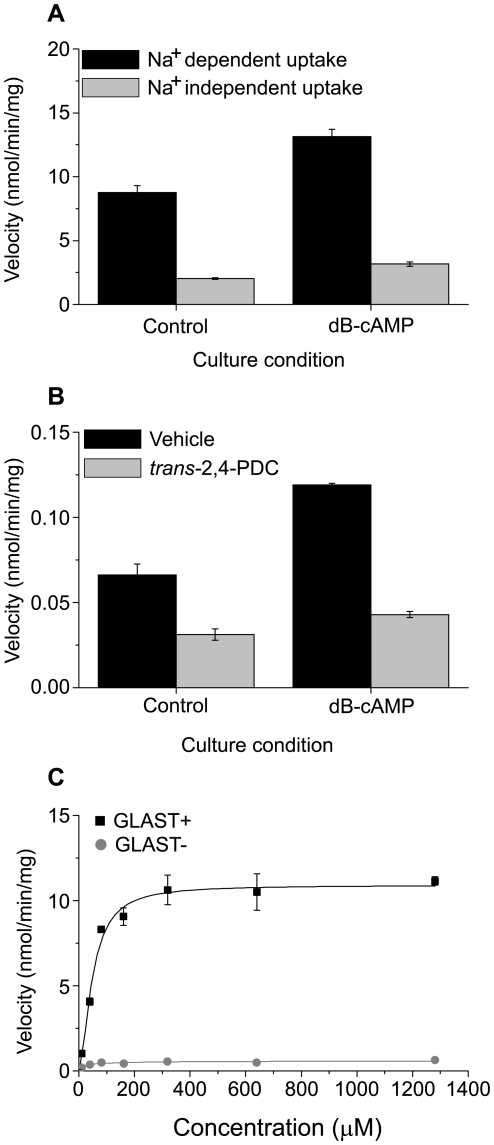
Glutamate uptake in rat cortical astrocytes depends on sodium and a high affinity transporter. A, Glutamate uptake in sodium-free buffer (LiCl) was significantly lower than in culture medium with sodium (n = 3, mean±SEM). Uptake was initiated with 500 µM glutamate. B, *Trans*-2,4-PDC, a high-affinity transporter inhibitor (100 µM), reduced glutamate uptake (mean±SEM, n = 3) by 55–65%. Uptake was initiated with 1 µM glutamate. C, Glutamate uptake was 18 times higher in cultures immunopositive for GLAST (GLAST+). All cells which expressed GLAST also expressed GFAP. Separate cultures of cells immunonegative for GLAST (GLAST-) were also immunonegative for GFAP and did not uptake glutamate (n = 3 cultures per cell type and concentration, mean±SEM).

We assessed the specificity of our glutamate assay in rat astrocytes by three independent methods (Fig. 8). We found that replacing NaCl with LiCl in the uptake buffer reduced glutamate uptake by approximately 75% regardless of whether the astrocytes were cultured with or without dB-cAMP for 10 days (p<0.001 for both conditions, Fig. 8A). Consistent with previous observations [22], we also found that 100 µM *trans*-2,4-PDC, a high-affinity glutamate transport inhibitor [22], [23], reduced uptake of 1 µM glutamate by 53% (p<0.01) for control and by 64% (p<0.00001) for astrocytes cultured for 10 days with dB-cAMP (Fig. 8B). Finally, we compared glutamate uptake between our astrocyte cultures and cells immunonegative for the astrocytes-specific markers glial fibrillary acidic protein (GFAP) and GLAST. We found that our astrocyte cultures imported significantly more glutamate at all doses tested (Fig. 8C). We conclude that the glutamate uptake we measured in cultured cortical astrocytes was mediated primarily by a high-affinity, sodium-dependent glutamate transporter which was constitutively active across the circadian day.

## Discussion

We found that astrocytes in culture take up glutamate as a function of two transcription factors (*Clock* and *NPAS2*) and one transcriptional regulator (*Per2*) associated with circadian function. We also found that addition of B27 or dB-cAMP to the culture medium increased expression of the clock genes, *Period1* and *Period2*, and also increased glutamate uptake. However, while cultured astroglia show high amplitude circadian rhythms in *Period* gene expression, we found no evidence for circadian changes in their glutamate uptake. We also failed to detect rhythms in glutamate reuptake in mouse cortical slices. This provides the first demonstration of a non-circadian role for clock genes in glial physiology. We conclude that, while it can be dynamically modulated by clock gene expression, glutamate uptake by cultured astroglia is not circadian.

How do CLOCK, NPAS2 and PER2 proteins modulate glutamate uptake? We found that glutamate uptake was reduced in glia homozygous for the *Clock* mutation or the *NPAS2* null mutation, indicating that their gene products augment glutamate uptake. Both CLOCK and NPAS2 have been shown to dimerize with BMAL1 protein to transcriptionally activate genes with E-box sequences in their promoter, such as *Per2* [12], [24], [25]. Thus, CLOCK or NPAS2 could increase glutamate uptake by activating transcription of genes involved in glutamate uptake. One candidate target gene would be *Glast*, the major transporter of glutamate in cultured astrocytes. Although the *Glast* promoter does not contain the canonical E-box associated with CLOCK/BMAL1 regulation [16], we found reduced *Glast* mRNA and protein levels in *Clock/Clock* and *NPAS2−/−* astroglia. CLOCK and NPAS2 could regulate *Glast* transcription indirectly, or GLAST protein stability or localization, events implicated in GLAST-mediated glutamate uptake [26]. Alternatively, the PER2 protein could be responsible for the regulation of GLAST and glutamate uptake. In this case, mutations in CLOCK and/or NPAS2 would reduce PER2 levels and decrease glutamate uptake. Importantly, there is no evidence for circadian modulation of glutamate uptake or GLAST function in astroglia indicating that CLOCK, NPAS2 and PER2 are constitutive activators of GLAST activity.

*Clock/Clock* mutant animals display several interesting phenotypes in addition to overt loss of circadian rhythms in locomotor activity [8], [27]. These include mania-like behaviors [9], obesity [28], [29] and increased dopaminergic and behavioral responses to cocaine [30]. It is tempting to speculate that reduced glutamate uptake by astrocytes in *Clock/Clock* mutants could contribute to their enhanced locomotor and physiological responses to cocaine, behaviors and neuroplastic changes in which a glutamatergic component has been demonstrated [31].

We can not exclude the possibility that the sensitivity of the assays we used failed to detect a low amplitude circadian rhythm of glutamate uptake. Supplementing the culture medium with B27 or dB-cAMP increased glutamate uptake by 13% or 39%, respectively. Importantly, these increases were detectable and significant. Thus, circadian modulation of glutamate uptake, if it exists, must be less than a 13% change. Rat astrocytes do show detectable, but relatively small changes (3–13%) in peak-to-trough *Per1*-driven circadian bioluminescence depending on the culture conditions. Mouse astrocytes showed a 140% change in peak-to-trough PER2 expression, but no detectable rhythm in glutamate uptake. Thus, it is possible that the amplitude of the oscillation of the circadian clock in cultured glia does not support rhythms in glutamate uptake.

It remains an intriguing possibility that glutamate uptake is circadian *in vivo* where astrocytes receive signals from other circadian cell types. It is possible, for example, that neurons enhance the ability of astrocytes to display circadian rhythms in glutamate uptake. Without neurons, astrocytes in culture do not express the GLT-1 high affinity glutamate transporter [32] which is the primary glutamate transporter in cortical astrocytes *in vivo* [33], [34]. However, we found no rhythms in glutamate uptake in cortical slices or in glia exposed to dB-cAMP, a treatment that induces GLT-1 expression and other changes similar to those produced by adding neurons to the culture [19], [32]. We conclude that the critical role of glutamate clearance by astroglia is not coupled to their circadian clock, but could be modulated by circadian signals from other cells.

## Materials and Methods

### Primary Astrocyte Culture

All procedures were approved by the Animal Care and Use Committee (Washington University, St Louis) and conformed to National Institutes of Health guidelines. All efforts were made to minimize the number of animals used, and their suffering. Unless otherwise noted, all of the reagents were purchased from Sigma (St. Louis, MO). We used transgenic *Per1::luc* rats (Japanese Wistar; founders a generous gift from Dr. H. Tei, MITILS, Tokyo, Japan) expressing 6.7 kb of the mouse *Per1* promoter driving firefly luciferase [35], homozygous knock-in mice (congenic on C57Bl/6) expressing *mPeriod2::luciferase* (PER2::LUC) fusion protein [36] and *Clock/Clock* mutant mice [37] (both founders generous gifts of Dr. J. Takahashi, Northwestern University, Evanston, IL). We also used homozygous neuronal PAS domain 2 deficient mice (*NPAS2−/−*, congenic on C57Bl/6, founders generous gift of S.L. McKnight, Univ Texas Southwestern, Dallas, TX [24], and mice with homozygous mutations in both the *Per1* and *Per2* genes (*mPer1^ldc^mPer2^ldc^*, here referred to as *Per1^m^Per2^m^* founders generous gift of Dr. S.M. Reppert, University of Massachusetts Medical School, Worcester, MA [14]. We generated *Per2^m^* homozygote mice by backcrossing *Per1^m^Per2^m^* founders with C57Bl/6 to generate F1 mice heterozygote for both *Per1* and *Per2*. We then inter-crossed F1 mice to generate F2. Genotype of F2 offspring was confirmed by PCR and only homozygote *Per2^m^* were used for cell culture. Cortical glial cultures were generated from neonates (postnatal days 2–6) following methods published previously [4], [38]. Astrocytes harvested from cultures that had been passaged between one and three times were plated into 6- or 12-well tissue-culture plates (glutamate uptake and immunocytochemistry) or into 35 mm Primaria-treated culture dishes (bioluminescence recordings, Falcon BD Biosciences, CA). Approximately 6.5×10^4^ or 1.5×10^5^ cells were plated per well (12-well and 6-well plates respectively) and grown until confluence (∼7–14 days). Full medium exchanges were performed twice weekly. In some experiments, we induced stellation of astrocytes through the addition of 250 µM dibutyryl cAMP (dB-cAMP) for 10 days. For all cultures, Time 0 denotes the time when we exchanged the medium on the day prior to measuring glutamate uptake and bioluminescence.

### Cortical Slice Preparation

Cortical slices were prepared according to the method of Abe et al (2002) [39] with modifications. Young (15–25 day old) PER2::LUC mice were anesthetized with CO_2_ and decapitated either 1–2 h before lights off (ZT10 group) or before lights on (ZT23 group). Their brains were dissected and placed in cold oxygenated HBSS in a vibroslicer. We made 300 µm sections and collected 2 mm cortical punches from the primary somatosensory cortex. Cortical punches were placed on a square piece of Millicell-CM organotypic culture membrane placed on top of a Millicell insert (Millipore, 0.4 µm pore size, PICM0RG50). The insert was placed into a 35 mm sterile Petri dish (Falcon BD Biosciences, CA) containing 1 ml of bioluminescence recording medium (see below). The dishes were sealed with vacuum grease and cultured for 36 h before glutamate uptake.

### Bioluminescence Recording

We monitored bioluminescence as previously published with slight modifications [4], [39]. *Per1::luc* or PER2::LUC activity was recorded in 6 min bins with a photomultiplier tube from cortical glia plated into a Primaria-treated 35 mm Petri dish with 1 ml of recording medium comprised of DMEM supplemented with 10% FBS, pen/strep, 10 mM HEPES, 0.35 g/L NaHCO3, 4.5 g/L D-glucose, 2% L-glutamine, 1x B27 cell culture supplement (Gibco, Carlsbad, CA) and 0.1 mM beetle luciferin (Promega, Madison, WI). Bioluminescence was recorded for at least 4 days.

### Glutamate Uptake

We performed glutamate uptake on parallel cultures to those used for bioluminescence recording using published methods [16]. We replaced the recording medium with uptake buffer (5 mM Tris base, 10 mM HEPES, 140 mM NaCl, 2.5 mM KCl, 1.2 mM CaCl_2_, 1.2 mM MgCl_2_, 1.2 mM K_2_HPO_4_, 10 mM Dextrose and 1 mM Methionine Sulfoximine) for 30 min. Methionine Sulfoximine was added to inhibit glutamine synthetase activity. After pre-incubation, we did a full buffer exchange with uptake buffer supplemented with radioactive glutamate for 10 min at 37°C. For circadian measurements, we added either 200 µM (mouse astrocytes) or 500 µM (rat astrocytes) glutamate with 0.25 µCi/ml of L-[G-3H]glutamate (55 Ci/mmol, GE Healthcare, Pittsburgh, PA) and for dose-response measurements, we added 1, 10, 40, 80, 160, 320, 640, or 1280 µM glutamate with 0.4 µCi/ml of L-[G-3H]glutamate. We terminated uptake with four wash buffer rinses (identical to uptake buffer except that NaCl was replaced by 140 mM LiCl) in 40 min at 4°C. We lysed the cells immediately after the fourth wash with 0.1 M NaOH and divided the lysate to measure glutamate levels by liquid scintillation counting (150–200 µl cell lysate, Wallac 1410 Liquid Scintillation Counter) and protein levels by the Bradford Assay (50 µl cell lysate). Sodium-dependent uptake was assessed in separate rat astrocyte cultures by replacing NaCl with LiCl in the uptake buffer. We also used L-*trans*-Pyrrolidine-2,4-dicarboxylic acid (*trans-*PDC, 100 µM, Tocris Bioscience, Ellisville, MO) in the uptake buffer to block glutamate uptake by high-affinity transporters (1 µM glutamate). We recorded uptake from triplicate cultures at each time point and glutamate concentration and reported their mean (±SEM) uptake velocity normalized to protein content (nmol/min/mg). For glutamate uptake from cortical punches, circadian time was estimated from the time of surgery with CT12 defined as the start of daily locomotor activity in the intact animal. Starting 36 h after *in vitro* preparation, cortical punches and their Millicell membrane were transferred to a 24-well culture plate and incubated for 30 min at 37°C in 300 µl of uptake buffer. We performed glutamate uptake in cortical punches using the same procedure as the pure astrocyte culture. We used 300 µl of uptake buffer containing 1, 10, or 25 µM glutamate with 0.2 µCi L-[G-3H]glutamate.

### Immunocytochemistry

We assessed the purity of replicate astrocyte cultures by double-immunostaining for the astrocyte-specific marker, glial fibrillary acidic protein (GFAP), and the high-affinity glutamate transporter, GLAST. Cells were washed three times in 0.01 M Phosphate Buffered Saline (PBS), fixed for 30 min in cold 4% paraformaldehyde, and washed again 3 times in PBS. Cells were then permeabilized for 30 min with 0.25% Triton X-100 in PBS (Triton-PBS) supplemented with 10% bovine serum albumin (BSA) and then incubated overnight at 4°C in a solution of 0.25% Triton-PBS with 3% BSA and a combination of a guinea-pig polyclonal anti-GLAST primary antibody (1∶2000 dilution, Chemicon, Catalog No. AB1782) and a rabbit polyclonal anti-GFAP antibody (1∶1000 dilution, DakoCytomation Denmark, Code No. Z0334). Astrocytes were then washed three times in PBS and incubated for 90 min at room temperature with a combination of a donkey anti-rabbit CY2 and a donkey anti-guinea-pig CY3 secondary antibody (1∶200 dilution for each, Jackson Immunoresearch, West Grove, PA). Astrocytes were then washed three times in PBS and cell nuclei were stained with 300 nM 4′,6- diamidino-2-phenylindole, dilactate (DAPI, Molecular Probes, Eugene, OR). Fluorescence images were captured with a Retiga-EX camera (Qimaging, Burnaby, British Columbia, Canada) at 10× magnification (TE200; Nikon, Melville, NY) using the Northern Eclipse software (Empix Imaging, North Tonawanda, NY). We found that immunostaining was absent when the primary antibodies were omitted. There was also no cross reactivity between the secondary antibodies. We also compared GLAST protein expression between *Clock/Clock*, *NPAS2−/−*, Per1*^m^*Per2*^m^* homozygous mutants and PER2::LUC homozygous (or wild-type) astrocytes by quantifying fluorescence as grayscale intensity (ImageJ software, W.S. Rasband, NIH Bethesda MD, http://rsb.info.nih.gov/ij/). The grayscale intensity of 6 non-overlapping fields (10× magnification) was measured and divided by the number of cells present in the field to yield a normalized fluorescence intensity for each culture. We determined cell density for each culture from DAPI-labeled nuclei in the same fields as above.

*qRT-PCR-* To study the effects of genotype on *Glast* expression, we extracted total RNA from *Clock/Clock* and PER2::LUC astrocyte cultures 24 h after a full medium exchange (n = 2 cultures per genotype, in three separate replicates of each experiment). To test whether there was a circadian rhythm in *Glast* expression, we extracted total RNA from PER2::LUC astrocytes, starting 24 h after a medium change, every 6 h up to 66 h after medium change (n = 2–3 cultures per time point) in TRIzol according to manufacturer's instructions (Invitrogen, Carlsbad, CA). qRT-PCR for *Glast* and *Gapdh* was performed using the Superscript III Platinum SYBR Green One-Step qRT-PCR Kit (Invitrogen, Carlsbad, CA) with TAQ enzyme and SYBR Green fluorescent detection according to manufacturer's protocol. For each sample, qRT-PCR was performed in triplicate on 50 ng of total mRNA using the following primers: *Glast*: Forward: 5′-CTGTTTCGGAATGCCTTCGTT-3′, Reverse: 5′-TCACCTCCCGGTAGCTCATTT-3′; *Gapdh*: Forward: 5′-AGCCTCGTCCCGTAGACAAAA-3′, Reverse: 5′-TGGCAACAATCTCCACTTTGC-3′. *Glast* mRNA was normalized to *Gapdh* mRNA abundance. Mean (±SEM) fold change in *Glast* expression for *Clock/Clock* astrocytes was calculated using the ΔΔCt (deltadeltaCt) method and expressed as a function of wild-type values. Mean (±SEM) fold change in *Glast* expression over time was expressed relative to the first time point.

### Data analysis

Glutamate uptake was analyzed by two-way analysis of variance (ANOVA) with medium composition and time since medium change as the independent variables. Maximal uptake velocity (Vmax) and concentration yielding half-maximal uptake (Km) for dose-response curves were determined by vertical nonlinear least squares (v-NLLS) regressions using LMMpro software (Alfisol LCC, C.P. Schulthess, University of Connecticut). Bioluminescence amplitude and rhythmicity were analyzed as described previously [4], [39]. Detrended bioluminescence was adjusted to the raw value of the first peak for each culture to compare bioluminescence levels and amplitude between different culture conditions. We measured mean bioluminescence levels for the second 24 h portion of the recording.
